# 

**Published:** 1880-08

**Authors:** 


### INDEX.

### A

PAGE.
Abnormal Obturator Arteries, Fatal Herniotomy, due to, . . . . 73
Abscess of the Antrum of Highmore Simulating Acute Inflammation of the Lachrymal Sac, by B. H. Grove, M. D. 343
Accidents in Thoracentesis by Aspiration 173
Acetabulum, Fracture of the, . . . 68
Addison's Disease, by Thos. F. Rochester, M. D., . . . . . . . . . . 1
Albumen in the Urine, by Chas. A. Doremus, M. D. . . . . . . . . 440
Alumni Association, Buffalo Medical College, . . . . . . . . . 327
Albuminuria, Recent Studies in, by H. S. Kilbourne, M. D., . . . . . 54
Alcohol in the Atmosphere, . . . 508
Amputation, Primary and Secondary, . 220
Atmosphere, Alcohol in the, . . . 508
Anasarca, Apocynum Cannabinum in, . 507
Anasarca, Treatment of, . . . . . 502
Aneurism of the Innominate Artery, . 71
Angular Curvature of Spine, preceded by Lumbar Abscess, by Bernard Bartow, M. D., . . . . . . . . 299
Antiseptic Midwifery, . . . . . 319
Antiseptic Transfusion of Human Blood in a Patient, the Subject of Secondary Hæmorrhage, . . . . . . . . 273
Antiseptics in Ophthalmology, from the Spanish by Lucien Howe, M. D., . . . 119
Apocynum Cannabinum in Anasarca, . 507
Are Measles more dangerous in Grown-up Persons than in Children? Translated from the Danish by Herman Mynter, M. D., . . . . . . . 163
Are Most Women Crazy? . . . . . 235
Arrow Wounds, by H. S. Kilbourne, M. D., . . . . . . . . . . 538
PAGE.
Arsenic, How it Poisons, . . . . . 165
Arteria Anguli Nasi, Abnormal Position of the, by Lucien Howe, M. D., . . . 495
Artificial Nourishment, . . . . . 314
Artificial Respiration in New-born Children, . . . . . . . . . . 468
Asthma, Petrolium in, . . . . . 83
Atropine in Whooping-Cough, . . . . 85

### B

Barker, A. M., M.D., on Strangulation of the Small Intestine by the Mesentery of the Ileum, . . . . . . . . 117
Bartow, Bernard, M. D., on Sole-Leather Splints, . . . . . . . . . 108
Bartow, Bernard, M. D., on the Treatment of Rotary-Lateral Curvature of the Spine, . . . . . . . . . 481
Bartow, Bernard, M. D., on Angular Curvature of Spine, preceded by Lumbar Abscess, . . . . . . . . 299
Bichloride of Mercury in Dysentery and Diarrhœa, . . . . . . . . . 41
Blood-stains, easy Methods of Detecting, 150
Blood, Transfusion of, . . . . . 221
Bone, Lead Plate for a Portion of the Frontal, . . . . . . . . . 77
Bone-Setting, Natural, . . . . . 360
Breast-Cancer, Neglected Symptom in, . 268
Breast, Excision of Tumor of the, by Chas. C. F. Gay, M. D., . . . . . 159
Buffalo Medical and Surgical Association, . . . . . . . . . . 130
Buffalo Medical College, . . . . . 138
Buffalo Medical and Surgical Association, . . . . . . . . . . 178
Buffalo Medical College, . . . . . 182
Buffalo Medical Association, . . . 472

PAGE.

Buffalo State Asylum for the Insane, . 137
Buffalo State Asylum for the Insane, . 43
Buffalo State Asylum for the Insane, . 181

**C**

Calculi Biliary, Olive Oil for, . . . 211
Cancer of the Breast, Statistics of 250 cases of, . . . . . . . . . 69
Cancer of Liver and Pancreas, by Thos. Lothrop, M. D., . . . . . . . . 303
Cannabis Indica in Hemicrania, . . 322
Cary, Charles, M. D., on A Successful Case of Direct Tranfusion for Pernicious Anæmia, . . . . . . . 259
Catgut Ligature, . . . . . . . 453
Chancrous Folliculitis of the Vulva, . 37
Children, Hernia in, . . . . . . 227
Chloroform Death—Bill for Twelve Months, . . . . . . . . . 317
Chloroform Poisoning, Strychnia and Whiskey Hypodermically in, . . 504
Cholagogues, Recent Researches on the Action of, by H. S. Kilbourne, M. D., . 433
Choroiditis in Relapsing Fever. Translated from the German by Lucien Howe, M. D., . . . . . . . . 205
Clinic at Buffalo General Hospital, . . 414
Clinic Medical at the Buffalo General Hospital, . . . . . . . . . 346
Clinic Surgical at the Buffalo General Hospital, reported by F. Peterson, M.D. 296
Color Blindness, . . . . . . . 508
Constipation, Infantile, . , . . 288
Constitutional Syphilis, Second attack of, 82
Contraction of Fingers, . . . . . 38
Cooke, Henry P., M. D., on Urethral Chancroid Cicatrical Stricture, . . 8
Consumption, Treatment of, . . . 128
Cremation, by Frederick Peterson, M. D. 380
Curvature of the Spine, on the Treatment of Rotary Lateral, by Bernard Bartow, M. D., . . . . . . . 481
Cystitis, Treatment of, . . . . . 505

**D**

Dayton, Chas. L., M. D., on Foreign Bodies in the Stomach, . . . . 202
Determination of the Sexes, . . . 368
Digestive Ferments, . . . . . . 17
Digestion or Iron, . . . . . . . 39
Diphtheria, New Remedy in, . . . 222
Diphteria and Scarlatina, the Spread of, 283

PAGE.

Diphtheria, Treatment of, . . . . . 125
Dislocation of the Hip, by Herman Mynter, M. D., . . . . . . . 61
Doctors, The Forthcoming Harvest of, . 325
Doremus, Chas. A., on Albumen in the Urine, . . . . . . . . . . 440
Dysentery and Diarrhœa, Bichloroide of Mercury in, . . . . . . . 41

**E**

Ectropion by Transplantation of Skin, by Lucien Howe, M. D., . . . . . 398
Eczema at the Umbilicus, . . . . 323
Elbow Joint, Resection of, . . . 412
Empyema as a Result of Pleuro-Pneumonia. Translated from the Spanish by F. Peterson, M. D., . . . . . 64
Enteric Fever, . . . . . . . . 471
Erie County Medical Society, . . . 522
Erie County Medical Society, . . . 286
Erie County Medical Society, Semi-Annuel Meeting of the, . . . . . . 562
Erysipelatous Pneumonia, Translated from the French by F. Peterson, M. D., 16
Ethylate of Sodium in the Treatment of Nævus, . . . . . . . . . 459
Eustachian Tube, Injections of Medicated Fluids through the, Translated from the German by Lucien Howe, M. D., . . . . . . . . . , 449
Excellent Appointment, . . . . . 286
Excision of Tumors of the Breast, by Chas. C. F. Gay, M. D., . . . . . 159

**F**

Fat, Digestion of, . . . . . . . 21
Fatty Heart, by H. R. Hopkins, M. D., . 241
Fingers, Contraction of, . . . . . 38
Flatulence by Glycerine, . . . . 176
Foreign Bodies in the Stomach, by Chas. L. Dayton, M. D., . . . . . . 202
Food Adulteration, . . . . . . 503
Foetal Monstrosities, Maternal Shock in the Production of . . . . . . 25
Fowler, Joseph, M. D., on Statistics Relating to a Scarlatina Epidemic, . . 337
Fowler, Joseph, M. D., on Typhoid Fever, Complicated with Epistaxis and Tetanus, . . . . . . . . . 155
Fowler's Solution, Subcutaneous Injection of, . . . . , . . . . 40

PAGE.

Fracture of the Acetabulum, . . . . 60
Fracture of the Neck of the Femur, Pathognomonic Sign of, . . . . . 219
Fracture of the Skull, Compound Comminuted, . . . . . . . . . 350
Friction in Insomnia, from the French by F. Peterson, M. D., . . . . . 15
Frontal Bone, Compound Comminuted Fracture of the, by James S. Smith, M. D., . . . . . . . . . . 306

**G**

Gastric Ulcer and Cancer, . . . . . 316
Gay, Chas. C. F., M. D., on Excision of Tumors of the Breast, . . . . . 159
Germ Theory, One Phase of the, . . . 557
Glycerine in Flatulence, . . . . . 176
Gonorrhœa, New Method of Arresting, . 229
Gonorrhœal Rheumatism, by Herman Mynter, M. D., . . . . . . . . 488
Gout, Treatment and Pathology of, . 506
Granger, Wm. D., M. D., on State Regulation of the Practice of Medicine, . 98
Grove, B. H., M. D., on Abscess of the Antrum of Highmore Simulating Acute Inflammation of the Lachrymal Sac, . 343
Gunshot Wound of the Crural Artery. Translated from the German by Herman Mynter, M. D., . . . . . . 263

**H**

Health Reports, . . . . . . . 44
Heart, Valvular Lesions of the, . . 72
Heart, Milk Diet in Diseases of the, . 281
Heath, W. H., M. D., on The Heatonian Method for the Radical Cure of Hernia, 405
Heatonian Method for the Radical Cure of Hernia, by W. H. Heath, M. D., . 405
Hemicrania, Cannabis Indica in, . . 322
Hernia, the Heatonian Method for the Radical Cure of, by W. H. Heath, M. D., 405
Hermiotomy, Fatal, Due to Abnormal Obturator Arteries, . . . . . . 73
Hernia in Children, . . . . . . 226
Hip, Dislocation of the, by Herman Mynter, M. D., . . . . . . . 61
Hopkins, H. R., M. D., on Fatty Heart, . 241
Howe, Lucien, M. D., Translation from the Spanish on Antiseptics in Ophthalmology, . . . . . . . . 119
Howe, Lucien, M. D., on Strictures of the Nasal Duct, . . . . . . . . 49
Howe, Lucien, M. D., Translation on Choroiditis in Relapsing Fever, . . 205
Howe, Lucien, M. D., on Ectropion by Transplantation of Skin, . . . . 398
Howe, Lucien, M. D., Translation on Injections of Medicated Fluids through the Eustachian Tube, . . . . . 449
Howe, Lucien, M. D., on Unusual Position of the Arteria Anguli Nasi, . . 495
Human Blood in a Case of Leukæmia, Microscopical Examination of, Translated from the Dutch by P. W. Van Peyma, M. D., . . . . . . . . 498
Hyoscyamia in Insanity, . . . . . 75

**I**

Impotence from Salycilate of Soda, . . 515
Induction of Labor in Protracted Pregnancy, by P. W. Van Peyma, M. D., . 104
Inebriates, What shall we do with, . . 470
Infantile Constipation, . . . . . 280
Injection of Medicated Fluids through the Eustachian Tube, Translated from the German by Lucien Howe, M. D., . 449
In Memoriam, . . . . . . . . 92
In Memoriam, . . . . . . . . 424
Insane Asylums, . . . . . . . 378
Insane, Buffalo State Asylum for the, . 43
Insanity, Hyoscyamia in, . . . . . 75
Incision of the Tunica Vaginalis, . . 548
Innocuousness of Nitrous Oxide Gas, Translated from the Dutch by P. W. Van Peyma, M. D., . . . . . . 209
Innominate Artery, Aneurism of the, . 71
Intestinal Obstruction, or Occlusion, lasting thirty-nine days, . . . . , 276
International Medical Congress. . . 381
Intoxication in Shock during Operations, the Prevention of, . . . . . . 311
Intra-Uterine Medication, . . . . . 170
Iodide of Potassium in the Elimination of Lead, . . . . . . . . . 167
Iodoform as a Dressing. Translated from the German by Frederick Peterson, M. D., . . . . . . . . . 552
Iron on Digestion, . . . . . . 39

**J**

Julius F. Miner, Sickness of, . . . . 136

PAGE.

**K**

Keene, J. W., M. D., on A Case of Traumatic Tetanus, . . . , . . . 255
Keene, J. W., M. D., on A Case of Puerperal Eclampsia, . . . . . . 203
Kidney Movable, Translated from the Danish by Herman Mynter, M. D., . 164
Kilbourne, H. S., M. D., on Recent Studies in Albuminaria, . . . . . 54
Kilbourne, H. S., M. D., on Recent Researches on the Action of Cholagogues, 433
Kilbourne, H. S., M. D., On Arrow Wounds, . . . . . . . . . 5[illegible]8

**L**

Labor, Induction of, in Protracted Pregnancy, by P. W. Van Peyma, M. D., . 104
Laceration of the Cervix Ut^ri as a Cause of Post-partum Hemorrhage. . . 84
Lead Plate for a Portion of the Frontal Bone, . . . . . . . . . . 77
Liebrich on Ozone, . . . . . . 509
Ligature of Left Carotid, Translated by Herman Mynter, M. D., . . . . . 13
List of Physicians Registered in the Clerk's Office of the County of Erie, . 183
Lothrop, Thos., M. D., on Cancer of Liver and Pancreas, . . . . . . . 303
Lothrop, Thos., M. D., on Migraine, . 194
Lothrop, Thomas, M. D., On Notes on Puerperal Cases, . . . . . . 529

**M**

Maternal Shock in the Production of Fœtal Monstrosities, . . . . . 25
Medication, Intra-Uterine, . . . . 170
Medical Department of the University of Buffalo, . . . . . . . . 369
Medical Education, Statistics of, in the United States, . . . . . . . 364
Medical Law, The Enforcement of the, . 282
Medical Law, Its Enforcement, . . 328
Medical Law, The New, . . . . . 135
Medical Legislation, . . . . . . 90
Metric System in Medicine, . . . 225
Mesmerism, . . . . . . . . . 66
Micrococcus of Bluish Pus, Translated from the Dutch by P. W. Van Peyma, M. D., . . . . . . . . . . 14
Midwifery, Antiseptic, . . . . 319
Migrame, by Thos. Lothrop, M. D., . . 194

PAGE.

Milk Curdling Ferment, . . . . . 19
Milk Diet in Diseases of the Heart, . 281
Misuse of Iron Preparations, . . . 174
Movable Kidney, Translated from the Danish by Herman Mynter, M. D., . 164
Municipal Appointments, Health Department, . . . . . . . . 285
Mynter, Herman, M. D., on Gonorrhœal Rheumatism, . . . . . . . 488
Mynter, Herman, M. D., on Stricture of the Trachea, . . . . . . . 289
Mynter, Herman, M. D., Translation on Movable Kidney, . . . . . . 164
Mynter, Herman, M. D., Translation on Are Measles more Dangerous in Grown-up Persons than in Children, . 163
Mynter, Herman, M. D., Translation on Gunshot Wound of the Crural Artery, 263
Mynter, Herman, M. D., on Dislocation of the Hip, . . . . . . . . 61
Mynter, Herman, M. D., Translation on Ligature of Left Carotid. . . . 13
Mynter, Herman, M. D., on Nephrolithiasis, Nephrotomy, . . . . . . 111

**N**

Nævus, Ethylate of Sodium in the Treatment of, . . . . . . . . 459
Nasal Duct, Strictures of the, by Lucien Howe, M. D., . . . . . . . 49
Natural Bone-Setting, . . . . . . 360
Neglected Symptom in Breast-Cancer, . 268
Nephrolithiasis, Nephrotomy, by Herman Mynter, M. D., . . . . . . 111
Nephritic Colic, Treatment of, by Chas. G. Stockton, M. D., . . . . . 145
Nephrotomy, Nephrolithiasis, by Herman Mynter, M. D., . . . . . . 111
Neuralgia, Tonga in, . . . . . . 38
Neuralgia Treated with Tonga, . . 81
New Medical Law, The Registration under, . . . . . . . . . 328
New-Born Children, Artificial Respiration in, . . . . . . . . . 468
New York State Pharmaceutical Association, . . . . . . . . . . 516
Nitrous-Oxide Gas, Innocuousness of, Translated from the Dutch by P. W, Van Peyma, M. D., . . . . . . 209
Nitro-Glycerine, The Therapeutical Value of, . . . . . . . . 168

PAGE.

O

Obstinate Vomiting in Pregnancy, . . 507
Olive Oil, Biliary Calculi, . . . . . 211
Our Public Institutions, . . . . . 519
Optic Nerve, Spinal Root of the, . . 320
Ophthalmology, Antiseptics in, Translation from the Spanish by Lucien Howe, M. D., . . . . . . . . . . 119
Ovariotomy, Thomas Keith and, . . 79
Ovariotomy, A Case of Double, . . 549
Ozone, Liebrich on, . . . . . . 509

P

Pathognomonic Sign of Fracture of the Neck of the Femur, . . . . . . 219
Peterson, Frederick, M. D., on Puerperal Septicemia, . . . . . . 114
Peterson, F., M. D., Translation on Friction in Insomnia, . . . . . . 15
Peterson, F., M. D., Translation on Erysipelatus pneumonia, . . . . 16
Peterson, Frederick, M. D., Translation on Empyema as a Result of Pleuro-Pneumonia, . . . . . . . . 64
Peterson, F., M. D., on Surgical Clinic at the Buffalo General Hospital, . . 296
Peterson, Frederick, M. D., on Cremation, . . . . . . . . . . 385
Peterson, Frederick, M. D., on Iodoform as a Dressing, . . . . . . . 552
Pepsin and Trypsin, . . . . . . 17
Peptized Milk, . . . . . . . . 219
Perineum Ruptured, . . . . . . 12
Perineorrhaphy, An Improved method of, 30
Pernicious Anæmia, successful Case of Direct Transfusion for, by Charles Cary, M. D., . . . . . . . 259
Petroleum in Asthma, . . . . . 83
Pharmacal Failings, . . . . . . 231
Phosphate of Lime, . . . . . . 39
Physicians, List of, Registered in the Clerk's Office of the County of Erie, . 183
Placenta, Adhesion of the, . . . . . 465
Pleuro-Pneumonia, Empyemia as a Result of, Translated from the Spanish by F. Peterson, M. D., . . . . . . 64
Poisionous Drugs, The Sale of, . . . . 475
Positive Sign of Pregnancy During the First Three Months, . . . . . . 271
Post Partum Hemorrhage, Laceration of the Cervix Uteri as a Cause of, . . 84

PAGE.

Positive Sign of Pregnancy During the First Three Months, . . . . . . 123
Pregnancy During the First Three Months, Positive Sign of, . . . . 123
Pregnancy, Positive Sign of, During the First Three Months, . . . . . . 271
Pregnancy, A Case of Extra-Uterine, by Prof. James P. White, . . . . . 446
Pregnancy, Obstinate Vomiting in, . 507
Preparations, Misuse of Iron, . . . 174
Pressure in Traumatic Aneurism, . . 218
Primary and Secondary Amputation, . 220
Puerperal Cases, Notes on, by Thomas Lothrop, M. D., . . . . . . . . 529
Puerperal Eclampsia, A Case of, by J. W. Keene, M. D., . . . . . . 203
Puerperal Septicemia, by Frederick Peterson, M. D., . . . . . . 114
Pylorus, Extirpation of the . . . 513

Q

Quinine with Nervous Sedatives, . . 321

R

Recent Studies in Albuminuria, by H. S. Kilbourne, M. D., . . . . . . 54
Reflex Cough, . . . . . . . . 321
Registered Practitioners, . . . . . 423
Relapsing Fever, Choroiditis in, Translated from the German by Lucien Howe, M. D., . . . . . . . . 205
Rochester, Thomas F., M. D., on Addison's Disease, . . . . . . . . 1
Royal Infirmities, . . . . . . . 382
Rupture of Axillary during Reduction of Luxation of the Humerus . . . 215
Ruptured Perineum, . . . . . . . 12
Rupture of the Uterus, Translated from the German by P. W. Van Peyma, M. D., . . . . . . . . . . 307

S

Salicylate of Soda, Impotence from, . 513
Scarlatina Epidemic, Statistics Relating to a, by Joseph Fowler, M. D., . . 337
Scarlatina and Diphtheria, The Spread of, 283
Second Attack of Constitutional Syphilis, 87
Sexes, Determination of, . . . . . 368
Sloughing and Destruction of the Anus and Rectum, . . . . . . . . 9
Smith, Jas. S., M. D., on Compound Comminuted Fracture of the Frontal Bone, 306

PAGE.
Sole-Leather Splints, by Bernard Bartow, M. D., . . . . . . . . . 108
Specific Agent of Typhoid Fever, . . 322
Spencer Wells, . . . . . . . . 89
Spinal Root of the Optic Nerve, . . 320
Splints, Sole-Leather, by Bernard Bartow, M. D., . . . . . . . . . 108
Statistics of Medical Education in the United States, . . . . . . . 364
State Regulation of the Practice of Medicine, by William W. Granger, M. D., . 98
Stockton, Charles G., M. D., on Treatment of Nephritic Colic, . . . 145
Stomach, Foreign Bodies in the, . . 202
Strangulation of the Small Intestine by the Mesentery of the Ileum, by A. M. Barker, M. D., . . . . . . . 117
Stricture of the Trachea, by Herman Mynter, M. D. . . . . . . . . 289
Strictures of the Nasal Duct, by Lucien Howe, M. D., . . . . . . . 49
Stricture of the Urethra, Treatment of, . 227
Strychnia and Whiskey Hypodermically in Chloroform Poisoning, . . . 504
Sub-Involution of the Uterus, Notes on the Treatment of, . . . . . 31
Subcutaneous Injection of Fowlers' Solution, . . . . . . . . . 40
Successsful Case of Direct Transfusion for Pernicious Anæmia, by Chas. Cary, M. D., . . . . . . . . . . 259
Summer Diarrhœa, Etiology of, . . 23
Superfœtation, A Case of, . . . . . 273
Suppurative Pleuritis, Translated from the German by P. W. Van Peyma, M. D. 352
Surgical Cases, . . . . . . . 545
Suturing Divided Nerve Trunks, . . 34

T

Trachea, Stricture of the, by Herman Mynter, M. D., . . . . . . . 289
Traumatic Aneurism, Pressure in, . . 218
Traumatic Tetanus, A Case of, by J. W. Keene, M. D., . . . . . . . 255
Transplanation of a Testicle from the Groin to the Scrotum, . . . . 214
Transfusion of Blood, . . . . . . 221
Transplantation of Skin, Ectropium by, by Lucien Howe, M. D., . . . . 398
Travers vs. Boardman, . . . . . 356
Treatment of Rupture of the Uterus, . 217
Treatment of Stricture of the Urethra, . 227
Treatment of Nephritic Colic, by Charles G. Stockton, M. D., . . . . . . 145
Temperance Cause, Bad for the, . . 235
Tenia and Teniafuges, . . . . . 554
The Buffalo Medical College, . . . 477
Therapeutic Review for 1880, . . . 365
Thoracentesis, Accidents in, by Aspiration, . . . . . . . . . . 173
Thomas Keith and Ovariotomy, . . 79
Tight Rings, . . . . . . . . 505
Tonga, Neuralgia Treated with, . . 82
Tonga in Neuralgia, . . . . . . 38
Trypsin and Pepsin, . . . . . . 17
Tuberculosis in Dogs, Translated from the German by P. W. Van Peyma, M.D., 418
Twentieth Volume, . . . . . . 42
Typhoid Fever, Specific Agent of, . 322
Typhoid Fever, Complicated with Epistaxis and Tetanus, by Joseph Fowler, M. D., . . . . . . . . . . 155

U

Ulcer and Cancer, Gastric, . . . . 316
Umbilicus, Eczema at the, . . , 323
Urethral Chancroid, Cicatrical Stricture, by Henry P. Cooke, M. D., . . . 8
Urine, Albumen in the, by Chas. A. Doremus, M. D., . . . . . . . 440
Uterine Diseases, . . . . . . . 463
Uterus, Treatmənt of Rupture of the, . 217

V

Valvular Lesions of the Heart, . . 73
Van Peyma, P. W., M. D., Translation on Tuberculosis in Dogs, . . . . 418
Van Peyma, P. W., M. D., Translation on Suppurative Pleuritis, . . . . 352
Van Peyma, P. W., M. D., on Rupture of the Uterus, . . . . . . . . 307
Van Peyma, P. W., M. D., Translation of Innocuousness of Nitrous Oxide Gas, . 209
Van Peyma, P. W., M. D., Translation on Micrococcus of Bluish Pus, . . . 14
Van Peyma, P. W., M. D., on Induction of Labor in Protracted Pregnancy, . 104
Van Peyma, P. W., M. D., Translation on Microscopical Examination of Human Blood in a Case of Leukæmia, . 498
Vivisection once more, . . . . 421
Vulva, Chancrous, Folliculitis of the, . 37

W

White, Prof. James P., on A Case of Extra-Uterine Pregnancy, . . . 446

